# Biological functions of the Olig gene family in brain cancer and therapeutic targeting

**DOI:** 10.3389/fnins.2023.1129434

**Published:** 2023-05-18

**Authors:** Jenny I. Szu, Igor F. Tsigelny, Alexander Wojcinski, Santosh Kesari

**Affiliations:** ^1^Department of Translational Neurosciences, Providence Saint John’s Health Center, Saint John’s Cancer Institute, Santa Monica, CA, United States; ^2^San Diego Supercomputer Center, University of California, San Diego, San Diego, CA, United States; ^3^CureScience, San Diego, CA, United States; ^4^Pacific Neuroscience Institute, Santa Monica, CA, United States

**Keywords:** Olig, transcription factor, cancer, glioblastoma, glioma, medulloblastoma, leukemia, melanoma

## Abstract

The *Olig* genes encode members of the basic helix–loop–helix (bHLH) family of transcription factors. Olig1, Olig2, and Olig3 are expressed in both the developing and mature central nervous system (CNS) and regulate cellular specification and differentiation. Over the past decade extensive studies have established functional roles of *Olig1* and *Olig2* in development as well as in cancer. Olig2 overexpression drives glioma proliferation and resistance to radiation and chemotherapy. In this review, we summarize the biological functions of the Olig family in brain cancer and how targeting Olig family genes may have therapeutic benefit.

## Introduction

### The Olig family

The oligodendrocyte lineage transcription factor (Olig) family of proteins, comprised of Olig1, Olig2, and Olig3, are basic helix–loop–helix (bHLH) transcription factors that are essential regulators of neural cell fate and specification (reviewed in detail in Szu et al., 2021). The Olig genes and the proteins they encode are responsible for the development of oligodendrocytes and neural cells. Initial studies revealed that the Olig genes, primarily *Olig1* and *Olig2*, are expressed by oligodendrocyte precursor cells (OPCs) where *Olig1* regulates oligodendrocyte formation and maturation in the brain and *Olig2* modulates oligodendrogenesis in the spinal cord (Lu et al., 2002; Ross et al., 2003; Meijer et al., 2012). It was later discovered that the Olig proteins are also involved in neurogenesis. Specifically, Olig2 is distinctly expressed in the progenitors of motor neurons (pMN) domain of the developing spinal cord where motor neurons are generated (Takebayashi et al., 2000; Novitch et al., 2001). Furthermore, Olig1/2 were also found to generate inhibitory interneurons in the brain (Miyoshi et al., 2007; Silbereis et al., 2014). Recently, GABAergic neurons were shown to be derived in Olig3 lineage cells (Lowenstein et al., 2021). The role of the Olig family in astrocyte specification is not well established. It was shown that Olig1/2 may promote astrocyte differentiation where astrocytes are sequentially transformed after generation of interneurons (Zhou and Anderson, 2002). However, other studies determined that Olig-expressing precursors to be negative regulators of astrocytes confirming the cellular fate to be neurons and oligodendrocytes (Lu et al., 2002). The Olig genes are also expressed in cancer and are detailed below.

### Gliomas

Gliomas are primary brain tumors derived from neuroglial stem cells or progenitor cells as well as lineage restricted precursors (Weller et al., 2015). Roughly 30% of all primary brain tumors are gliomas and 80% are considered malignant (Schwartzbaum et al., 2006; Weller et al., 2015). Traditionally, gliomas are classified based on their histopathological and clinical features established by the World Health Organization (WHO; Louis et al., 2021). Over the years, technological advancement has vastly improved classification of gliomas centered on molecular and genomic changes (Reifenberger and Collins, 2004). Currently, gliomas are divided into six families: (1) adult-type diffuse gliomas; (2) pediatric-type diffuse low-grade gliomas; (3) pediatric-type high-grade gliomas; (4) circumscribed astrocytic gliomas; (5) glioneuronal and neuronal tumors; and (6) ependymomas (Louis et al., 2021). Adult-type diffuse gliomas are further separated into three different groups: (1) *isocitrate dehydrogenase* (*IDH*) mutant with 1p/19q co-deletion with primarily oligodendroglial morphology; (2) *IDH* mutant with 1p/19q non-codeletion with mainly astrocytic histology; and (3) *IDH* wildtype (Louis et al., 2021). Gliomas are also further categorized based on WHO grades I–IV. Grade I gliomas consists of pilocytic astrocytic astrocytomas and are primarily observed in the cerebellum or brain stem of children. Grade II gliomas are comprised of adult low-grade gliomas and are generally astrocytomas, oligodendrogliomas, mixed oligoastrocytomas, and diffuse astrocytomas. Grade III gliomas are malignant gliomas composed of anaplastic cells. Grade IV gliomas are glioblastoma (GBM) which consists of primary and secondary GBM (Louis et al., 2021).

### Olig expression in gliomas

Success in prognosis and therapeutics of gliomas is dependent on accurate diagnosis. While there exist a multitude of histological markers to differentiate between the types of gliomas, challenges remain due to gliomas displaying similar morphological characteristics (Collins, 2013). Specifically, reliable markers to accurately distinguish between glioma types are lacking due to ambiguous histological features. Differentiating gliomas based on their cellular morphology can also be confusing and biased, resulting in classifications such as oligoastrocytoma or mixed glioma (Brennan, 2011). Observer variability can also lead to misdiagnosis or underdiagnosis of the correct disease (Marie et al., 2001).

CNS tumors are heterogenous and their grading based on histological features is notoriously subjective (Theeler et al., 2012; Komori, 2021). With advances in diagnostic technologies, the most recent WHO 2021 classification of tumors of the CNS have adopted key molecular markers and revised grading of astrocytic tumors, oligodendroglial tumors, oligoastrocytomas, glioneuronal tumors, and neuronal tumors (Louis et al., 2021). The summary of Olig expression in gliomas presented in this review is based on WHO CNS tumor classification at the time the studies were conducted. However, we have organized the gliomas, to the best of our ability, centered on the most current 2021 WHO CNS tumor classification. Large datasets have confirmed the expression of Olig2 across all gliomas such as TCGA and others (Singh et al., 2004; Suva et al., 2014).

#### Olig expression in adult-type diffuse gliomas

Adult-type diffuse gliomas are composed of *IDH*-mutant and 1p/19q-codeleted oligodendrogliomas, *IDH*-mutant astrocytomas, and *IDH*-wildtype GBMs, based on histological characteristics and explicit molecular markers. In adults, oligodendrogliomas with *IDH* mutation and 1p/19q-codeletion also present with mutations in the *TERT* promoter gene (Lee et al., 2018). Oligodendroglial tumors have generated great interest over the past decade due to its favorable response to chemotherapy (Engelhard et al., 2003; Reifenberger and Louis, 2003; Van den Bent et al., 2008) which may be attributed to the concurrent loss of chromosome arms 1p and 19q (Cairncross et al., 1998; Smith et al., 2000; Sasaki et al., 2002). *IDH*-mutant astrocytomas are now graded as WHO grade II, III, or IV (Louis et al., 2021) and also harbor *ATRX* and *TP53* mutations (Marker et al., 2021). GBM is the most common and deadliest primary brain tumor. *IDH*-wildtype GBM demonstrates alterations in epidermal growth factor receptor (EGFR), and similar to oligodendrogliomas, exhibit *TERT* promoter mutations (Galbraith et al., 2020).

Because oligodendrogliomas arise from oligodendrocytes, it is not surprising that attempts to diagnose oligodendrogliomas have utilized oligodendrocyte markers. Mature oligodendrocyte markers, such as myelin basic protein (MBP) and proteolipid protein (PLP), however, are not expressed at detectable levels in oligodendrogliomas (Sung et al., 1996; Popko et al., 2002). Furthermore, immature oligodendrocyte markers, such as the chondroitin sulphate proteoglycan NG2 and platelet-derived growth factor receptor alpha (PDGFR-α), lack specificity (Popko et al., 2002) and have been unsuccessful in discerning between glioma types (Shoshan et al., 1999; Marie et al., 2001). Several earlier studies have observed marked *Olig2* expression in oligodendrogliomas (Lu et al., 2001; Marie et al., 2001; Yokoo et al., 2004). Specifically, anaplastic oligodendrogliomas displayed intense nuclear *Olig2* expression (Ohnishi et al., 2003). Morphologically, *Olig* positive cells were moderately to densely packed, and displayed round and homogeneous nuclei with perinuclear halos (Lu et al., 2001), characteristics consistent with oligodendroglial tumors (Kim et al., 2005). Others have also observed an upregulation of both *Olig1* and *Olig2* in these tumors (Ohnishi et al., 2003; Aguirre-Cruz et al., 2004; Riemenschneider et al., 2004). For example, one study found an astounding 87% (26/30) and 93% (28/30) of oligodendroglial samples were positive for *Olig1* and *Olig2*, respectively (Aguirre-Cruz et al., 2004). Furthermore, the strong expression of *Olig1* and *Olig2* was shown to be correlated to WHO classification with their expression increasing incrementally from grades I to III (Ohnishi et al., 2003). However, one report did note varied expression of *Olig1* and *Olig2*. Here, the authors found 3 grade III oligodendrogliomas did not express either *Olig1* or *Olig2* while another 3 grade III oligodendrogliomas expressed *Olig1* only (Bouvier et al., 2003).

Compared to oligodendrogliomas, Olig expression in astrocytomas and GBMs has been inconsistent and varied. Generally, low levels of *Olig1* and *Olig2* have been observed (Ohnishi et al., 2003; Riemenschneider et al., 2004; Tanaka et al., 2008) with weak *Olig2* intensity in the nuclei (Ohnishi et al., 2003). In one report, low *Olig1* expression was detected along with a marked upregulation of *Olig2* (Riemenschneider et al., 2004), while another study found an upregulation of both *Olig1* and *Olig2,* although the sample size was small (4 cases of diffuse astrocytomas; Aguirre-Cruz et al., 2004). In another study astrocytomas were found to exhibit only weak or moderate *Olig* expression (Lu et al., 2001). Olig expression was not detected in a case of grade III astrocytoma (Bouvier et al., 2003). GBMs also displayed varying *Olig2* expression. While one study rarely observed *Olig2* in GBM (Ohnishi et al., 2003), another study demonstrated lower mean transcript levels of *Olig1* and *Olig2* (Riemenschneider et al., 2004). In one rare case of GBM, upregulation of both *Olig1* and *Olig2* were observed (Aguirre-Cruz et al., 2004). Interestingly, in a separate study, Olig2 protein levels were upregulated in all cases of GBM and appeared nuclear (Ligon et al., 2004).

#### Olig in pediatric-type diffuse high-grade gliomas

While diffuse high-grade gliomas (HGGs) are more common in adults, pediatric HGGs present with similar histopathological features and devastating prognosis (Jones and Baker, 2014; Wu et al., 2014). Pediatric diffuse HGGs can arise from various regions in the brain but most develop as diffuse intrinsic pontine glioma (DIPG) which occurs in the brainstem (Jones and Baker, 2014) during a restricted window of childhood (median age ~ 7 years; Hennika and Becher, 2016). DIPGs are the most common brainstem tumors in children with a median of survival of less than 1 year from diagnosis (Warren, 2012). Histopathologically, DIPG hosts a spectrum of features that is consistent with diffuse and anaplastic astrocytomas and GBMs (Buczkowicz et al., 2014). Because DIPG appears during development, neural stem cells (NSCs) and neural progenitor cells (NPCs), which are actively proliferating and differentiating, are highly impacted during disease progression (Anderson et al., 2017). Olig proteins are critical players in cellular specification and differentiation during development (Szu et al., 2021). Their expression in DIPGs have been investigated. Not surprising, a large number of cells in the pons were found to be positive for Olig2 with a subset of these cells also co-expressing Sox2 and Nestin (Monje et al., 2011; Ballester et al., 2013), markers of not only CNS embryogenesis (Vinci et al., 2016), but also tumorigenesis (Boumahdi et al., 2014; Neradil and Veselska, 2015).

#### Olig in circumscribed astrocytic gliomas

Circumscribed astrocytic gliomas are astrocytic neoplasms with circumscribed growth (Riemenschneider et al., 2004). Pilocytic astrocytoma (PA) is a type of circumscribed astrocytic glioma and is considered a low-grade glioma (LGG). PAs occur mostly in children and young adolescent but can be observed in older patients as well. This brain tumor is commonly observed in the cerebellum, spinal cord, and optic pathways, but can occur anywhere in the brain (Riemenschneider et al., 2004; Ferris et al., 2017). Histologically, PAs display the classical biphasic pattern which is composed of compact areas containing Rosenthal fibers and loose microcystic areas (Ceppa et al., 2007).

*Olig* expression in PAs have been conflicting. Some studies have found low to moderate expression of *Olig1* and *Olig2* (Lu et al., 2001; Ohnishi et al., 2003) while others have reported high expression of these genes (Bouvier et al., 2003; Tanaka et al., 2008; Otero et al., 2011). One study observed greater immunoreactivity of Olig1 (97%; 62/64) compared to Olig2 (75%; 48/64; Takei et al., 2008). Diffuse staining patterns of Olig2 were observed (Otero et al., 2011) and similar to oligodendrogliomas, Olig immunoreactivity was found localized to the nuclei (Takei et al., 2008; Tanaka et al., 2008). Interestingly, double immunolabeling of Ki67 and Olig2 showed that most proliferating cells were also positive for Olig2, however, Ki67^+^ cells embodied a small portion of Olig2 expressing cells as PAs are LGGs and have a low rate of proliferation (Tanaka et al., 2008; Otero et al., 2011).

#### Olig in glioneuronal and neuronal tumors

Glioneuronal tumors (GNTs) are exceptionally rare neoplasms composed of both mixed neuronal and glial cells. The majority of GNTs are classified as grade I and are associated with seizures (Gatto et al., 2020; Krauze, 2021). The pathological aspects of GNTs remain unclear however, case reports have found Olig2 commonly expressed in these tumors and thus lean more toward oligodendrogliomas. Three subtypes of GNTs that demonstrate Olig2 upregulation are dysembryoplastic neuroepithelial tumors (DNTs; Komori and Arai, 2013; Matsumura et al., 2013), papillary glioneuronal tumors (PGNTs; Tanaka et al., 2005; Chen et al., 2006; Gelpi et al., 2007; Iżycka-Świeszewska et al., 2008; Matsumura et al., 2013), and rosette-forming glioneuronal tumors (Wang et al., 2009; Luan et al., 2010; Xiong et al., 2012; Matsumura et al., 2014).

DNTs are highly heterogenous with varying morphological features. Histologically, these tumors display nuclear atypia, mitosis, endothelial proliferation, or increased cell density, however, these appearances provide no prognostic value (Daumas-Duport et al., 1999). DNTs are also subtyped as simple or complex which displays oligodendroglia-like cells (OLCs) and floating neurons (Suh, 2015). With these hallmarks, the definition of DNTs remain controversial. DNTs were found to be more similar to oligodendrogliomas rather than a glioneuronal tumor. In this same study 88% of OLCs were diffusely Olig2^+^ and 10% of these cells also colocalized with galectin3 in the nuclei of OLCs. Few OLCs were positive for PDGFRα and did not exhibit 1p/19q codeletion. Additionally, NeuN^+^ and Olig2^+^ cells were mutually exclusive, further suggesting that DNTs are clear glial tumors rather than glioneuronal tumors (Komori and Arai, 2013).

Similarly, Olig2 expressing cells were also found in PGNTs suggesting that these tumors may be oligodendroglial or oligodendroglial-like. Histologically, PGNTs exhibit two distinct architectures: (1) pseudopapillary structures surrounded by (2) compact regions consisting of neuronal elements under different maturation stages (Tanaka et al., 2005; Gelpi et al., 2007; Iżycka-Świeszewska et al., 2008; Marumo et al., 2013).

### Origin of Olig2-positive glioma cells and cancer stem cells

Since the discovery of a small proportion of clonogenic progenitors in acute myeloid leukemia (AML; Griffin and Lowenberg, 1986), the existence of tumor initiating cells or cancer stem cells (CSCs) in several types of cancers were investigated. Thus arose the cancer stem cell hypothesis which states that malignant tumors are driven and sustained by a group of cells with stem cell properties of unlimited capacity for self-renewal and the ability to differentiate into any cell type (Reya et al., 2001; Singh et al., 2004). While the cancer stem cell hypothesis was adopted by many, it remains highly controversial (Bjerkvig et al., 2005; Jordan, 2009). Even with increasing evidence of CSCs, possibly the most debated aspect of the hypothesis is the mere existence of CSCs, as they only contribute to a small fraction of the tumor (Jordan, 2009; Konrad et al., 2017). Therefore, it remains unclear from where CSCs originated and, as a result, identifying a cell of origin in gliomas, such as GBMs, has been explored.

GBMs are the most common and aggressive primary malignant brain tumors. They exhibit a high degree of heterogeneity resulting in molecular subtypes of classical, mesenchymal, and proneural (Alifieris and Trafalis, 2015). Evidence of brain tumor initiating cells *in vivo* (Singh et al., 2004) have led to studies identifying glioma stem cells (GSCs) and better understanding of their properties. While markers such as CD133, CD15, L1CAM, CD49f, and SOX2 have been shown to be enriched in GSCs (Singh et al., 2004; Lee et al., 2006; Son et al., 2009; Lathia et al., 2010; Trépant et al., 2015) they are not exclusive to GSCs. Identification of more specific markers of GSCs could increase detection for developing targeted therapies. In one study, comparative analysis demonstrated *Olig2* as the most specific GBM stem cell marker (Trépant et al., 2015). Similar to previous findings (Ligon et al., 2004), Olig2 immunoreactivity was observed in all cases of GBM (Trépant et al., 2015) and was primarily nuclear with rare cases exhibiting cytoplasmic Olig2 staining. Further analysis revealed higher expression of Olig2 in secondary GBM compared to primary GBMs (Trépant et al., 2015). Secondary GBMs evolve from diffuse astrocytomas and have frequent *TP53* and *ATRX* mutations (Ohgaki and Kleihues, 2013; Marker et al., 2021) which are also commonly observed in *IDH-mutant* astrocytomas (Mirchia and Richardson, 2020). Because secondary GBMs were removed from the 2021 WHO CNS tumor classification, it is plausible that they are more closely related to *IDH*-mutant astrocytomas. Re-characterization of *Olig2* in “secondary GBM” samples is therefore necessary for thorough understanding of glioma pathogenesis.

Despite cell lineage studies, it is unclear why Olig2 is enhanced in oligodendrogliomas and astrocytomas (Kabel et al., 2018; Mallick, 2021). Olig2 is critical during CNS development. It is known for its role in oligodendrocyte and neuron specification and maturation and may also fulfill a potential function in astrocyte differentiation (Szu et al., 2021). In the postnatal brain, Olig2 functions as a repressor of neuronal lineages to direct subventricular zone (SVZ) progenitor cells toward astrocytic and oligodendrocytic fates (Marshall et al., 2005). In fact, overexpression of Olig2 in the SVZ increases the number of highly migratory OPCs to differentiate into mature oligodendrocytes (Maire et al., 2010). Neural stem cells (NSCs) in the SVZ, also known as Type B cells, are a subpopulation of GFAP positive astrocytes that give rise to neurons, astrocytes, oligodendrocytes, and NG2^+^ OPCs (Doetsch et al., 1999; Menn et al., 2006; Gonzalez-Perez et al., 2009; Gonzalez-Perez and Alvarez-Buylla, 2011). Interestingly, these Type B cells, along with some Type C (transit-amplifying) cells, also express Olig2 (Hack et al., 2005; Menn et al., 2006), indicating possible cells of origin for astrocytomas and oligodendrogliomas.

### Mechanisms underlying Olig2 expression in gliomas

Olig2 dysregulation in gliomas suggests that it is required for glioma growth and formation. Below we describe how Olig2 may be activated during cancer progression. Additionally, we explore how Olig2 drives gliomagenesis and whether it serves an oncogenic function.

#### Sonic hedgehog signaling activates Olig2 in gliomas

Throughout CNS development, NSCs and NPCs transform into distinct cell types in a spatiotemporal manner. A central function of Olig2 is to direct cell fate and specification, particularly into oligodendrocytes and neurons, in distinct regions of the brain and spinal cord during development (Szu et al., 2021). Olig2 is induced by Sonic hedgehog (Shh; Ortega et al., 2013) where its pathways are known to regulate cellular patterning and cell fates (Dessaud et al., 2008). The interplay between Shh and fibroblast growth factor (FGF) promotes Olig2 transcription (Tsigelny et al., 2016; Farreny et al., 2018). Increasing evidence has associated Shh signaling pathway with CNS tumors, however its relationship with Olig2 in gliomas is only beginning to be elucidated.

Several lines of evidence have associated Shh signaling with gliomas. For example, overexpression of Shh was observed in CD133^+^ cells and accelerated tumor growth while inhibition of Shh or shRNA knockdown of Shh delayed tumor growth and downregulated Ptch1 and Gli1 (Hung et al., 2020). Shh is activated via binding to the Ptch1 receptor while Gli1 is transcriptionally induced by Shh signaling (Cohen et al., 2015). Aberrant activation of Gli1 (Avery et al., 2021) and mutations in Ptch1 (Wang et al., 2019) are correlated with various cancers. In another study, expression of *Shh* and Ptch1 levels were significantly higher in brainstem astrocytomas compared to supratentorial astrocytomas (Yu et al., 2011). Increased levels of Notch receptors and its ligands were observed in astrogliomas and GBMs. Interestingly, glioma cell lines expressing the active form of Notch1 proliferated faster than those that did not (Zhang et al., 2008). Furthermore, overexpression of Notch1 further increased formation of Nestin^+^ neurosphere colonies (Zhang et al., 2008) and its expression in GBM cells (Shih and Holland, 2006). Similarly, overexpression of Notch1, its ligands, and downstream targets (*Hes1* and *Hes2*) have been detected in GBM. Notch activation has also been shown to contribute to Ras-mediated transformation of glial cells to glioma growth, proliferation, and survival (Kanamori et al., 2007).

Because Olig2 activity is regulated by Shh (Ortega et al., 2013), it is plausible that increased levels of Olig2 in gliomas are contributed by Shh deregulation. Recently, Olig2 was shown to behave as an oncogenic activator in Shh medulloblastoma (Shh-MB; Zhang et al., 2019), a malignant pediatric brain tumor characterized by activation of Shh signaling (Skowron et al., 2021). Olig2^+^ progenitors were identified as the rapidly dividing Type C cells at the onset of tumorigenesis. Surprisingly, a substantial increase in Olig2^+^ progenitors was found in recurrent Shh-MB indicating that Olig2^+^ progenitors are reactivated during recurrence or metastasis. Finally, enhanced Olig2^+^ expression was also detected in Shh-MB and was significantly correlated with decreased survival.

#### EGFR signaling activates Olig2

Studies have also illustrated Olig2 participation in positive feedback loops with the EGFR receptor tyrosine kinase (RTK; Kupp et al., 2016; Tsigelny et al., 2016). EGFR signaling is known to activate the oncogenic PI3K-AKT–mTOR and RAS–RAF–MEK–ERK pathways (Asati et al., 2016). Exposure to EGF leads to proliferation of Olig2^+^ type C cells (Hack et al., 2005; Menn et al., 2006; Ligon et al., 2007) and inhibition of EGFR signaling results in Olig2 depletion indicating that EGFR signaling is responsible for sustaining Olig2 expression in progenitor cells (Kupp et al., 2016). Furthermore, Olig2 directly targets *EGFR* (Meijer et al., 2014; Mateo et al., 2015) and overexpression of Olig2 leads to significant upregulation of *EGFR* and transcripts (Kupp et al., 2016). Additionally, phosphorylated Olig2 leads to differentially regulated genes associated with RTKs (Meijer et al., 2014; Kupp et al., 2016).

#### Downstream signaling effects of Olig2

**Figure 1:**
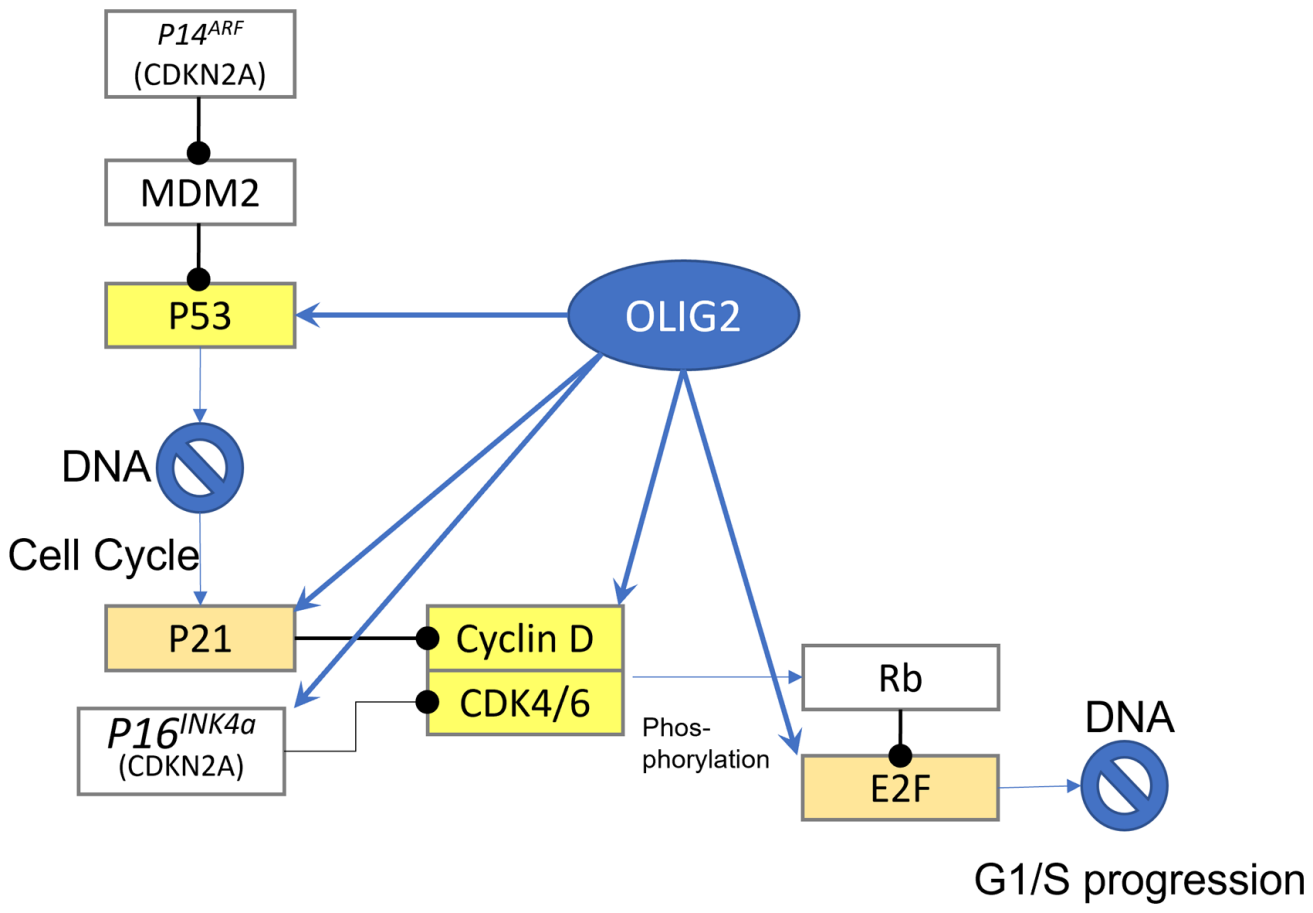
Gene targets of Olig2. Highlighted yellow are genes that can be bound by olig2 in the promoter-TSS region, light brown highlighted genes can be bound in more distant area before gene sequence.

**Figure 2:**
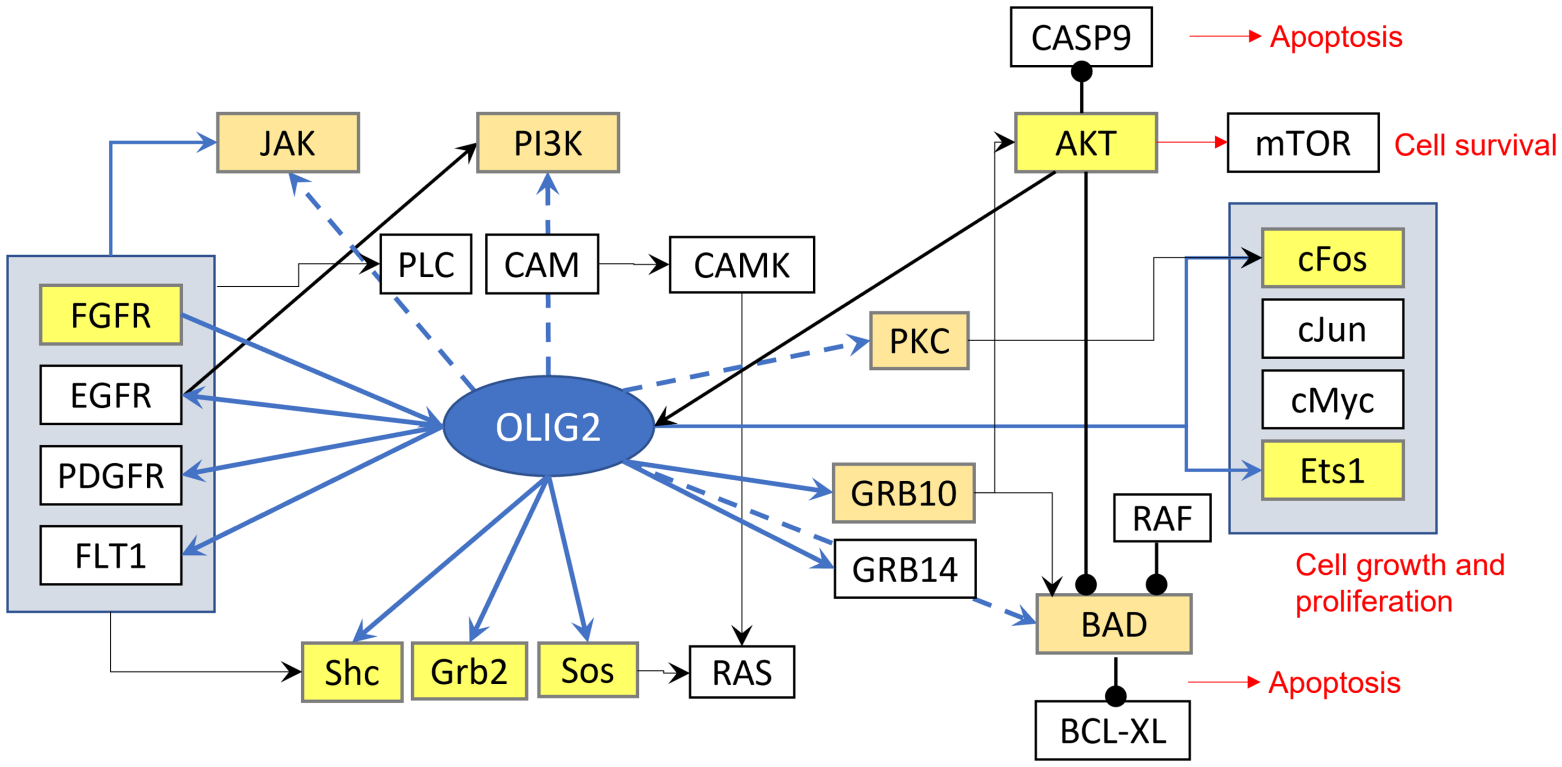
Signaling effects of Olig2. Highlighted yellow are genes that can be bound by olig2 in the promoter-TSS region, light brown highlighted genes can be bound in more distant area before gene sequence.

Gene network analysis has identified potential roles of Olig2 involvement in gliomas (Figures 1, 2). One such network entails cell cycle regulation (Tsigelny et al., 2016). *p53* is a tumor suppressor gene that functions in growth arrest and apoptosis in response to cellular stress. An effector of *p53* and cell cycle inhibitor is p21 (Haupt et al., 1997). Chromatin immunoprecipitation (ChIP) analysis demonstrated that p21 is a direct target of Olig2 repression in NPCs and gliomas (Ligon et al., 2007). Malignant gliomas that are resistant to radiation and genotoxic drugs are associated with reduced p53 functions as a result of Olig2 expression. However, in the absence of Olig2, even attenuated p53 functions were shown to be sufficient to activate radiation-induced apoptosis and growth arrest. Olig2 opposes p53 functions by suppressing acetylation of p53. Therefore, Olig2 acts as post-translational modifier of p53 to repress its downstream biological activities (Mehta et al., 2011).

### Olig2 directed treatment for gliomas

It appears that Olig2 may be an actionable drug target as multiple gliomas express high levels of Olig2. Additionally, several studies utilizing transgenic mouse models showed that ablation of *Olig2* delayed tumor growth and improved survival (Ligon et al., 2007; Mehta et al., 2011; Lu et al., 2016). Therefore, pharmacological inhibition of Olig2 may be therapeutically beneficial in treating gliomas.

In collaboration with Curtana Pharmaceuticals (San Diego, CA), we generated an orally bioavailable small molecule (397 kD) Olig2 inhibitor, CT-179, the first drug targeting bHLH transcription factors for cancer treatment. Our preliminary findings suggest that CT-179 prevents Olig2 homodimerization and strongly inhibited cellular growth and induced apoptosis of Olig2^+^ cells (Tsigelny et al., 2017). Moreover, CT-179 disrupts the cell cycle, ultimately resulting in mitotic catastrophe at the prometaphase. Treatment with CT-179 in tumor-bearing mice resulted in a reduction of Olig2^+^ cells and markedly improved survival outcome (Chen et al., 2017; Johns et al., 2018). Recent preliminary findings indicated that CT-179 also decreased Shh signaling and prolonged event-free survival in a mouse model of medulloblastoma (Dismuke et al., 2021).

Olig2 exhibits a dichotomous function. It displays a pro-neural function by promoting motor neuron differentiation as well as an anti-neural role by participating in generation of oligodendrocytes (Szu et al., 2021). Phosphorylation of Olig2 has been shown to regulate neuronal-glial cellular fate switch. Specifically, Olig2 was shown to be phosphorylated at serine 147 (S147) during motor neuron production (Li et al., 2011). Additionally, triple serine motif phosphorylation sites (S10, S13, S14) were shown to control proliferative functions of Olig2 (Sun et al., 2011). In fact, phosphorylated Olig2 exhibits pro-mitotic and anti-p53 functions (Mehta et al., 2011). Thus, targeting Olig2 or protein kinase inhibitors (PKIs) may have therapeutic effects against gliomas. Certainly, we observed that treatment with CT-179 decreased Olig2 phosphorylation in a mouse model of medulloblastoma (Dismuke et al., 2021) which may enable p53-mediated apoptosis (Mehta et al., 2011; Sun et al., 2011) and improve outcomes.

## Conclusion

The Olig proteins are members of bHLH transcription factors that modulate cellular fate. Specifically, Olig1 and Olig2 regulate neuron and oligodendrocyte development during brain and spinal cord development. Due to their specific roles in cellular specification, their expression has been examined in CNS tumors. Here, we explored the various types of gliomas that display marked upregulation of Olig mRNA or protein.

While observations of Olig2 expression have been conflicting in different types of gliomas, its upregulation was clearly indicated in oligodendrogliomas. This is not surprising as oligodendrogliomas arise from oligodendrocytes. Moreover, it seems gliomas that result from aberrations in OPCs or from regions of highly proliferative cells also tend to show an increase in Olig2 expression. DNTs and DIPGs are examples of such gliomas with marked Olig2 expression. Other gliomas, such as astrocytomas and GBMs, have varying Olig2 expression. Therefore, Olig2 may not be a specific marker for a distinct type of glioma.

Olig2 expression in other types of cancer is also plausible. For example, a recent study discovered upregulation of Olig2 in melanoma. Despite its hallmark nuclear staining observed in gliomas, Olig2 immunoreactivity in melanoma was predominantly cytoplasmic (Lee et al., 2021). It remains unclear what role Olig2 plays in melanoma. Olig2 overexpression has also been observed in leukemia. Interestingly, upregulation of Olig2 alone is weakly oncogenic in leukemia, however, together with *LMO1* and *Notch1,* overexpression results in cell proliferation (Lin et al., 2005). Because Olig2 levels were detected outside the CNS, its expression in other types of cancers warrants further investigation.

It appears that Olig2 may be a therapeutic target in gliomas. Small molecule inhibitors, such as CT-179, present as a promising strategy in targeting transcription factors for improving outcomes in brain cancer. Additionally, tyrosine kinase inhibitors may also present a beneficial therapeutic option as phosphorylated Olig2 seems to promote gliomagenesis. However, because Olig2 levels vary between different types of gliomas, thorough characterization of genetic targets of distinct glioma types are necessary for the identification of biomarkers and drug development. It is also concerning that Olig2, a CNS-restricted transcription factor, emerges in other cancer types outside the CNS. Therefore, investigation into Olig genes and proteins is urgently needed in all cancer types to determine how their activity influences disease outcome.

## Author contributions

SK conceived the manuscript. JS performed a complete literature review and drafted the manuscript and figures. IT, AW, and SK provided critical feedback on its content. IT drafted the figures. All authors contributed to the manuscript revision, read, and approved the submitted version.

## Funding

This work was supported by philanthropic gifts to Saint John’s Health Center Foundation.

## Conflict of interest

IT was patent holder and founder of Curtana Pharmaceuticals developing CT-179 for brain cancer. SK was patent holder, founder, advisor, and board member of Curtana Pharmaceuticals developing CT-179 for brain cancer.

The remaining authors declare that the research was conducted in the absence of any commercial or financial relationships that could be construed as a potential conflict of interest.

## Publisher’s note

All claims expressed in this article are solely those of the authors and do not necessarily represent those of their affiliated organizations, or those of the publisher, the editors and the reviewers. Any product that may be evaluated in this article, or claim that may be made by its manufacturer, is not guaranteed or endorsed by the publisher.
